# Editorial: Thymic Epithelial Cells: New Insights Into the Essential Driving Force of T-Cell Differentiation

**DOI:** 10.3389/fimmu.2021.744623

**Published:** 2021-08-13

**Authors:** Marita Bosticardo, Izumi Ohigashi, Jennifer E. Cowan, Nuno L. Alves

**Affiliations:** ^1^Laboratory of Clinical Immunology and Microbiology, IDGS, DIR, NIAID, NIH, Bethesda, MD, United States; ^2^Division of Experimental Immunology, Institute of Advanced Medical Sciences, University of Tokushima, Tokushima, Japan; ^3^Laboratory of Genome Integrity, Center for Cancer Research, National Cancer Institute, National Institutes of Health, Bethesda, MD, United States; ^4^i3S–Instituto de Investigação e Inovação em Saúde, Universidade do Porto, Porto, Portugal; ^5^IBMC–Instituto de Biologia Molecular e Celular, Universidade do Porto, Porto, Portugal

**Keywords:** thymus, thymic stromal cells, thymic epithelial cells, immunodeficiency, central tolerance

The thymus is the primary lymphoid organ specialized in the development of a diverse repertoire of non-self-reactive T lymphocytes. Thymic epithelial cells (TEC)s represent the main stromal cell type of the thymus regulating virtually all stages of T-cell development, from T-cell lineage commitment to positive, negative and T regulatory (Treg) cell selection (1, 2). These processes depend on the interaction between T-cell precursors with highly specialized subsets of TEC located within either the cortex; cortical (c)TECs, or medulla; medullary (m)TECs. While hematopoietic-intrinsic defects have been well characterized throughout the years, the study of TECs has been more complex, particularly in humans, due to their low cell density and difficulty in their isolation. Herein, we host a special issue focused on the latest discoveries in TEC biology.

Notably, proper TEC differentiation depends on reciprocal signals provided by developing thymocytes, a process commonly known as thymic cross-talk. Among the signals involved in thymic cross-talk, the interaction between the TNF receptor (TNFR) family member receptor activator of nuclear factor kappa-B (RANK), expressed by TECs, its ligand RANKL, expressed by positively selected CD4 thymocytes and a subset of group 3 innate lymphoid cells, controls mTEC proliferation/differentiation and TEC regeneration. In her review, Irla discusses recent advances in the complexity of the mTEC compartment, the role of the axis RANK/RANKL in TEC differentiation and regeneration and how the specific targeting of this axis may open the way to novel therapeutic approaches aimed at thymic regeneration and T-cell recovery.

Montero-Herradón et al. explored the minimal requirements of lympho-epithelial interactions to guarantee regular thymopoiesis. Using reaggregate thymic organ cultures comprising differing numbers of fetal TECs, grafted under the kidney capsule of FoxN1^-/-^ hosts, authors investigated how many TECs are necessary to support thymocyte maturation. The results demonstrated reduced T-cell-TEC cross-talk due to low TEC numbers is sufficient to support proper T-cell differentiation.

The establishment of central tolerance is a major function of the thymus and helps prevent the insurgence of autoimmune diseases. To establish and maintain central tolerance, elimination of self-reactive T lymphocytes through negative selection, and the generation of regulatory T-cells are required. mTECs and other antigen presenting cells, such as dendritic cells, are critical for both mechanisms. Throughout life the thymus undergoes conspicuous changes that affect the composition and function of TECs and antigen presenting cells, ultimately leading to alterations in central tolerance induction. Srinivasan et al. discuss how age-related changes to TECs and the thymic microenvironment affect T-conventional and T-regulatory cell selection and responses in humans and mice.

Autoimmune regulator (Aire)-expressing mTECs have an important role in tolerance induction. Indeed, the transcriptional regulator Aire is critical in inducing the expression of tissue-restricted antigens in mTECs, which direct self-reactive thymocytes toward apoptosis or Treg cell differentiation. In the last decade, several studies have indicated that mTECs continue to differentiate beyond Aire expression. However, the function and lineage relationships of these terminally differentiated post-Aire mTEC populations remain unclear. The perspective article by Laan et al. summarizes recent studies in mice and humans about these terminally differentiated mTEC subsets, including Hassall’s corpuscles structures, thought to be the final differentiation stage of the post-Aire lineage. Authors discuss their roles in tolerance induction, with a particular focus on their involvement in creating a pro-inflammatory microenvironment within the thymus.

One of the most important roles of TECs is to present MHC-associated antigens to developing thymocytes, in order to generate a wide repertoire of immunocompetent yet self-tolerant T-lymphocytes. Proteasomes degrade ubiquitinated proteins into peptide fragments that are eventually loaded onto MHC class I molecules. cTECs specifically express thymoproteasomes, which are essential for the positive selection of CD8+ T-cells, while other thymic cells, including mTECs, express immunoproteasomes that are critical for the elimination of self-reactive thymocytes. In their review article, Frantzeskakis et al. offer an overview of the functions and types of proteasomes expressed in the thymus, with a special focus on the most recent findings on thymoproteasomes and immunoproteasomes.

Although many aspects of TEC development and function have become increasingly clear in recent years, there are still several features of their basic biology that need further clarification. Biochemical studies on metabolic regulation of TECs have been especially challenging due to technical obstacles in TEC isolation. Semwal et al. discuss three distinct, but interconnected areas of TEC metabolic regulation: mTOR signaling, redox status of the cell, and autophagy. Authors discuss these three aspects in the establishment and maintenance of the thymic stromal compartment, along with age-associated dysfunctions.

Recent advances, particularly in scRNAseq, have added significant knowledge on the phenotypical and functional heterogeneity of TECs. However, there remains a lot of uncertainty about the identity of TEC progenitors (TECp) in the adult thymus. The prospective identification and isolation of adult TECp could present important clinical implications, as potentially they could be exploited to reverse age-associated alterations to the thymic environment or for the generation of thymic organoids in the field of regenerative medicine. In their review article, Ishikawa et al. discuss earlier and more recent studies that could help clarify the distinctive features of adult TECp, both in the homeostatic condition and recovery from thymic involution.

Another report on TECp and their age-related changes is provided by Pinheiro and Alves. The authors focus on the first weeks of murine post-natal life and highlight the timely coordination between the expansion and maturation of TECs and a presumable drop in the bioavailability of TECp during this specific period. Enhanced knowledge in this area offers strategies for reversal of thymic involution.

In addition to TECs, the thymic stromal cell compartment contains mesenchymal cells, including fibroblasts, pericytes, and endothelial cells. Two review articles in this issue discuss current and past knowledge on the function of these less studied, but important, non-epithelial thymic stromal populations. Nitta and Takayanagi consider historical studies and more recent advances in our understanding of the contribution of non-TEC stromal cells in thymic organogenesis and T-cell development. In particular, they highlight the role of fibroblasts on T-cell repertoire selection. James et al. discuss how in recent years, through improvements in the phenotypical identification and functional classification of fibroblasts and endothelial cells, their contribution to thymus development and function is beginning to be better understood.

This special issue on TECs includes original research articles presenting new insights into different phenotypical and functional aspects of TECs, ranging from *in-vitro* approaches, murine models and human samples. Gao et al. evaluate the role of the suppressor of cytokine signaling 3 (SOCS3), which is a negative regulator of cytokine signaling in thymic T-cell differentiation. Cytokines produced by both T-lymphocytes and TECs are crucial mediators for thymic cross-talk and are important for the development and maintenance of both stromal and lymphoid cells. Therefore, cytokine release and response need to be tightly controlled. Authors show that lack of SOCS3 leads to dramatic loss of thymic cellularity and altered cortico-medullary compartmentalization, resulting ultimately in a reduced output of recent thymic emigrants in SOCS3-deficient mice. Interestingly, they show that SOCS3 expression is mainly confined to TECs rather than thymocytes and identify a novel role for SOCS3 in supporting the maturation and anatomical distribution of TECs.

Tao et al. explore the role of TECs in the development of non- conventional, innate like T-cells, specifically iNKT and γδT-cells. This area of research remains much less well understood compared to the involvement of TECs in conventional αβT-cell differentiation. From the analysis of publicly available databases, authors found that transcripts of many cytokines and cytokine receptors are expressed by murine and human TECs. Particularly, they demonstrate that expression of IL-15 and IL-15Rα in TECs is crucial for thymic development of type 1 innate-like T-cells, such as iNKT1 and γδT1 cell, by showing a role for TECs not only as a source of IL-15 but also in the trans-presentation of IL-15 to ensure type 1 innate like T-cell development.

Han and Zúñiga-Pflücker explain the mechanism by which high oxygen in fetal thymus organ cultures (FTOCs) is critical to support efficient thymocyte development. It has been reported that fetal thymus lobes placed in low oxygen submersion (LOS) fail to support thymocyte development, whilst submersion cultures performed in the presence of high concentrations of ambient oxygen (ranging from 60 to 80%) (HOS) can facilitate normal thymocyte development. However, the mechanisms mediating the increased production of mature T-cells in HOS-FTOCs remained unknown. Here, authors demonstrate that HOS rescues the expression of FOXN1 and its target genes, DLL4 and CCL25, in addition to maintaining high levels of MHCII expression. Additionally, authors showed that increased oxygen availability can promote self-renewal of DN3 cells, resulting in an increased cellularity of submersion FTOCs.

Lastly, two articles from the group led by Villa and Bosticardo presented novel evidence of altered thymic development in Down Syndrome (DS) patients and in mice and patients with MHCII-deficiency. Based on previous studies suggesting immune senescence in DS, Marcovecchio et al., tested the hypothesis that induction of cellular senescence may contribute to early thymic involution and immune dysregulation in this syndrome. Authors showed that immunohistochemical analysis of thymic tissue in DS patients present with signs of accelerated thymic aging. Transcriptomic analysis of TECs revealed enriched expression of genes involved in cellular response to stress, epigenetic histone DNA modifications and senescence in DS samples. This signature was also confirmed in thymocytes and peripheral T-cells of DS patients. These results support a key role of cellular senescence and increased oxidative stress in the onset of immune dysregulation in DS patients. Ferrua et al. set out to explore the contribution of TEC alterations to the pathogenesis of MHCII deficiency, a rare combined immunodeficiency due to mutations in genes regulating the expression of MHCII molecules. The authors observed an overall perturbation of thymic structure and function in both MHCII^−/−^ mice and patients. Transcriptomic and proteomic profiling of TECs from MHCII^−/−^ mice revealed defective mTEC maturation and decreased promiscuous gene expression, causing defects in the establishment of central tolerance. Additionally, authors show peripheral tolerance impairment in MHCII deficiency, likely due to defective Treg cell generation and/or function and B-cell tolerance breakdown.

In summary, this special issue gives an overview of the up do date knowledge on TEC biology and their role in thymocyte development, in addition to providing novel research data that deepen our understanding about this fascinating and unique cell type.

## Author Contributions

All authors participated in the editorial reviews of the manuscripts. All authors participated in the writing and editing of the editorial article. All authors contributed to the article and approved the submitted version.

## Funding

M.B. is supported by the Intramural Research Program of the NIH. I.O. is supported by grant from the MEXT-JSPS 17K08884 and Takeda Science Foundation. The laboratory of N.L.A. is supported by a starting grant from the European Research Council (ERC) under the project 637843 and by FEDER - Fundo Europeu de Desenvolvimento Regional funds through the COMPETE 2020 - Operacional Programme for Competitiveness and Internationalisation (POCI), Portugal 2020, and by Portuguese funds through FCT - Fundação para a Ciência e a Tecnologia/Ministério da Ciência, Tecnologia e Ensino Superior in the framework of the project POCI-01-0145-FEDER-029129 (PTDC/MED-IMU/29129/2017) and PTDC/MED-IMU/1416/2020.

## Conflict of Interest

The authors declare that the research was conducted in the absence of any commercial or financial relationships that could be construed as a potential conflict of interest.

## Publisher’s Note

All claims expressed in this article are solely those of the authors and do not necessarily represent those of their affiliated organizations, or those of the publisher, the editors and the reviewers. Any product that may be evaluated in this article, or claim that may be made by its manufacturer, is not guaranteed or endorsed by the publisher.

## References

[1] Takahama Y Ohigashi I Baik S Anderson G. Generation of Diversity in Thymic Epithelial Cells. Nat Rev Immunol (2017) 17(5):295–305. 10.1038/nri.2017.12 28317923

[2] Kadouri N Nevo S Goldfarb Y Abramson J. Thymic Epithelial Cell Heterogeneity: TEC by TEC. Nat Rev Immunol Nat Res (2020) 20:239–53. 10.1038/s41577-019-0238-0 31804611

